# Alterations of Oxidative Stress Indicators, Antioxidant Enzymes, Soluble Sugars, and Amino Acids in Mustard [*Brassica juncea* (L.) Czern and Coss.] in Response to Varying Sowing Time, and Field Temperature

**DOI:** 10.3389/fpls.2022.875009

**Published:** 2022-05-03

**Authors:** Jyoti Chauhan, J. P. Srivastava, Rajesh Kumar Singhal, Walid Soufan, Basant Kumar Dadarwal, Udit Nandan Mishra, Hirdayesh Anuragi, Md Atikur Rahman, Mohamed I. Sakran, Marian Brestic, Marek Zivcak, Milan Skalicky, Ayman EL Sabagh

**Affiliations:** ^1^Department of Plant Physiology, Institute of Agriculture Sciences, Banaras Hindu University, Varanasi, India; ^2^Indian Council of Agricultural Research-Indian Grassland and Fodder Research Institute, Jhansi, India; ^3^Department of Plant Production, College of Food and Agriculture, King Saud University, Riyadh, Saudi Arabia; ^4^Faculty of Agriculture, Sri University, Cuttack, India; ^5^Indian Council of Agricultural Research-Central Agroforestry Research Institute, Jhansi, India; ^6^Grassland and Forage Division, National Institute of Animal Science, Rural Development Administration, Cheonan, South Korea; ^7^Biochemistry Section, Chemistry Department, Faculty of Science, Tanta University, Tanta, Egypt; ^8^Institute of Plant and Environmental Sciences, Faculty of Agrobiology and Food Resources, Slovak University of Agriculture, Nitra, Slovakia; ^9^Department of Botany and Plant Physiology, Faculty of Agrobiology, Food and Natural Resources, Czech University of Life Sciences, Prague, Czechia; ^10^Department of Agronomy, Faculty of Agriculture, Kafrelsheikh University, Kafrelsheikh, Egypt

**Keywords:** antioxidant defense, climate change, sowing time, fatty acid, oxidative stress, osmoprotectant

## Abstract

The impact of elevated temperature at the reproductive stage of a crop is one of the critical limitations that influence crop growth and productivity globally. This study was aimed to reveal how sowing time and changing field temperature influence on the regulation of oxidative stress indicators, antioxidant enzymes activity, soluble sugars (SS), and amino acids (AA) in Indian Mustard. The current study was carried out during the *rabi* 2017–2018 and 2018–2019 where, five varieties of mustard *viz*. Pusa Mustard 25 (PM-25) (V1), PM-26 (V2), BPR-541-4 (V3), RH-406 (V4), and Urvashi (V5) were grown under the field conditions on October 30 (normal sowing; S1), November 18 (late sowing; S2) and November 30 (very late sowing; S3) situations. The S1 and S3 plants, at mid-flowering stage, showed a significant variation in accumulation of SS (8.5 and 17.3%), free AA (235.4 and 224.6%), and proline content (118.1 and 133%), respectively, and played a crucial role in the osmotic adjustment under stress. The results showed that S3 sowing, exhibited a significant induction of the hydrogen peroxide (H_2_O_2_) (110.2 and 86.6%) and malondialdehyde (23.5 and 47.5%) concentrations, respectively, which indicated the sign of oxidative stress in plants. Interestingly, the polyphenol oxidase, peroxidase, superoxide dismutase, and catalase enzyme activities were also significantly increased in S3 plants compared to S1 plants, indicating their significant roles in ameliorating the oxidative stress. Furthermore, the concentration of fatty acid levels such as palmitic, stearic, oleic, and linoleic acids level also significantly increased in S3 plants, which influenced the seed and oil quality. The study suggests that the late sowing significantly impaired the biochemical mechanisms in Indian mustard. Further, the mustard variety V4 (RH-406) was found to be effective for cultivation as well as environmental stress adoption in Indian soils, and it could be highly useful in breeding for developing heat-tolerant genotypes for ensuring the food security.

## Introduction

The family Brassicaceae is regarded as one of the most economically important plant families (Friend, 2019). The genus *Brassica* consists over hundred species of rapeseed (*Brassica napus* L.), cabbage (*Brassica oleracea* L.), turnip rape (*Brassica rapa* L.), and mustard (*Brassica juncea* L.), which are predominantly cultivated for oil, vegetables, condiments, and fodder purposes (Raza et al., 2020). Currently, amid the dynamic abiotic constituents of the environment, the steadily and continuously ascending ambient temperature due to global warming, which is drastically influencing on plant growth, development, and productivity (Raza et al., 2019; Chand et al., 2022). In the Asian countries including India, where the major cultivated area is under rain-fed agro–ecosystem, the time of sowing solely depends on the rainfall availability. The rabi crops are important agricultural crops that are seeded in the winter season (last week of October to the mid-week of November) and harvested in spring (last week of April to mid-week of May) in India. Mustard is very sensitive to slight changes in moisture percentage and temperature during sowing, which affects the all stages of future plant development (Naylor et al., 2007; Ashish et al., 2019). Furthermore, the late sowing of rabi crops sets the crop for the high temperature and low moisture conditions during the reproductive and maturation stages, which are responsible for huge yield and quality losses (Patel et al., 2017; Yadav et al., 2020). Several studies have been focused on the impact of the late sowing conditions in mustard at different growth stages, and it is found that the late sowing negatively associated with the seed germination potential, growth, and development of leaf, root, and shoot, and physiological and biochemical processes. Moreover, the reproductive growth and development (pollen sterility, fertilization, and seed setting), and eventually the yield and quality attributes are drastically affected, when the mustard sowing is delayed (Godara et al., 2016; Bazzaz et al., 2020; Tripathi et al., 2020). Therefore, understanding the modification in antioxidant enzymes, and metabolites response under late sowing in mustard is crucial to reduce the yield gap potential (Sallam et al., 2018; Dawood et al., 2020).

Further, the heat stress manifestations are mediated through oxidative damage concerning to the generation of the reactive oxygen species (ROS). The ROS damages various biomolecules such as DNA, lipid, proteins, etc., and thus the damages fatally affect the plant metabolism and limit growth and yield (Mohan et al., 2020a; Javeed et al., 2021). Therefore, this auspice is conceiving a serious concern amide the scientists, as changes in environmental components such as elevated temperature have lethal consequences on plant life (Hassan et al., 2021). As these plants are sessile in nature and unable to move in a more favorable environments, the plant functional processes are substantially affected, often lethally, by elevated temperature (Raza, 2020; Ahmad M. et al., 2021). The primary mechanism of cellular membrane disruption under heat stress severely affects the photosynthetic and mitochondrial activities and decreases the ability of the plasma membrane to retain solutes (Chauhan et al., 2020; Dey et al., 2021). An elevated-temperature impairment can ensue in huge pre-harvest and post-harvest crop causalities. The plants alter the accumulation of metabolites in respect to elevated temperatures, especially by generating compatible solutes that can systematize macromolecules and cellular structures, retain turgidity of cell *via* osmotic adjustment, and remodel the antioxidant defense system to revive the homeostasis and cellular redox balance (Sallam et al., 2018; Mawlong et al., 2020; Mohan et al., 2020a).

The proline is an amino acid (AA) and an important indicator in determining the stress tolerance ability including heat. The accumulation of proline acts as osmoprotectants and worked as ROS quencher, which cope up the drastic effects of heat stress (Tonhati et al., 2020). Furthermore, it has been reported that the proline detoxifies the membrane due to the harmful ROS and protect the cell machinery by dysfunction due to the lipid peroxidation (Rajametov et al., 2021). The recent studies supported that the proline has numerous roles during stress as it improves the photosynthesis rate and also crosstalk's with numerous signaling molecules such as gasotransmitters (nitric oxide), and plant growth regulators, to activate stress signaling (Hanif et al., 2021; Rajametov et al., 2021; Singhal et al., 2021). Like proline, soluble sugar (SS) also acts as osmoprotectant and helps in the maintenance of the cellular redox balance, ROS detoxification, protection of photosynthetic apparatus, up-regulation of stress-related genes, and also acts as signaling molecule under stress condition (Ahmad F. et al., 2020; Afzal et al., 2021). To restrict the oxidative impairment, the plants also have a set of advanced detoxification systems, which transform the highly destructive ROS into non-toxic compounds (Mohan et al., 2020b). The plants shield the cells and sub-cellar systems damage from the ROS using antioxidant enzymes such as ascorbate peroxidase (APX), peroxidase (POX), superoxide dismutase (SOD), catalase (CAT), glutathione reductase (GR), polyphenol oxidase (PPO), and metabolites such as glutathione (GSH), ascorbic acid (AsA), tocopherol, and carotenoids (Rai A. N. et al., 2020). Substantial changes occur in antioxidant system linked to unfavorable environment, including the enzymatic and non-enzymatic antioxidants. The union of the antioxidant enzymes (SOD, CAT, and GR) and several others reduces the oxidative damages on the intracellular components (Raseeth et al., 2013; Ahmad Z. et al., 2020).

The total fatty acids (FAs) in the plant composed of unsaturated FA (PUFA), primarily, including the linoleic, linolenic and oleic acid, and saturated FA (palmitic and stearic acid) (Hu et al., 2018). These FAs are constituents of plasma membrane, provide the finite shape of the cell and help in the protection against unfavorable conditions (Rogowska and Szakiel, 2020). Saturation and composition of FA is associated with the stress tolerant ability in plants as they provide strength the cell membrane (Hu et al., 2018). The FA are the crucial biochemical attributes, determine the quality of seeds and oil in mustard crop. Heat stress also affects the saturation and unsaturation of FA through the membrane lipid peroxidation and modifying the organelle structure (Sinha et al., 2017; Higashi and Saito, 2019).

Therefore, considering these facts, the prime aim of this study is to examine the effects of different dates of sowing (normal, late, and very late) on oxidative stress indicators, antioxidant enzymes activity, and SS and AA contents in Indian mustard. Therefore, improving the seed yield of Indian mustard under late sowing conditions by genetic makeup and physiological scaling of elevate temperature tolerance at the reproductive stage would be crucial for the sustainability in oilseed production. In this investigation, our objective was to characterize the effects of high temperature on oxidative stress indicators and antioxidant enzymes to identify the genotypes that are tolerant to high temperatures, through their response to different sowing dates. This information is important to identify suitable gene donors to be used in the *Brassica* breeding program.

## Materials and Methods

### Sowing of Seed and Growth Conditions

The present investigation was carried with five varieties of Indian mustard [*B. juncea* (L.) Czern and Coss.] (PM 25; V1, PM 26; V2, BPR-541-4; V3, RH- 406; V4, Urvashi; V5) whose seeds were obtained from the Department of Genetics and Plant Breeding, Institute of Agricultural Sciences, BHU, Varanasi, India. These five varieties were sown in the field for two subsequent years (2017–2018, 2018–2019) on the dates of 30 October (S1), 15 November (S2), and 30 November (S3), under normal fertility conditions (Supplementary Figure S1) and standard agronomic practices (Chauhan et al., 2020). The physical, mechanical, and chemical properties of soil used during experimentation are highlighted in Supplementary Table S1.

The experimental farm situated in the Northern Gangetic Alluvial Plain at 25°18' N (North latitudes), 83°03' E (East longitude), and at an altitude of 128.93 m at mean sea level (MSL). The experimental plot was well-drained and an assured a source of sufficient water supply. The maximum and the minimum temperatures during 2017–2018 and 2018–2019 ranges between (6.34–19.17°C and 6.71–20.64°C) and 20.56–35.89°C and 21.40–37.56°C, respectively. The daily maximum and minimum temperatures (°C), relative humidity (%) (morning and evening), and rainfall (mm) during the crop growth period (1 November to 15 April) were obtained from the Institute Meteorology Section of the Department of Agronomy and shown in Supplementary Table S2. The mean relative humidity was near to 68%, which increased to 82% during the wet season and reduced up to 30% during the dry weather.

In the experimental field, the seeds were seeded in a factorial randomized complete block design (RCBD) with three replications. The 4-m length row was used, which had a row to row (R–R) distance of 45 cm and plant to plant distance (P–P) of 10 cm. Each plot had five rows, with a width of 2.25 m. The gross land area used for the present experiment was 585 m^2^ and the net sown area is 405 m^2^. The samples were collected from a separate experimental plot during the time of the investigation, and each row was selected randomly, leaving the border rows. Three random plants from each plot were selected for observations. The oxidative stress indicators [malondialdehyde (MDA) and H_2_O_2_ content], antioxidant enzymes activities [CAT (EC1.11.1.6), SOD (EC1.15.1.1), POX (EC1.11.1.7), PPO (EC1.14.18.1)] and SS, AA, proline content (PC), starch content (SC) and FA were recorded at 50% flowering.

### Determination of Oxidative Stress Indicators

The amount of H_2_O_2_ production was observed by spectrophotometer at the 50% flowering stage and represented as μmol g^−1^ fresh weight (FW). The H_2_O_2_ content (molar extinction coefficient 0.28 μM^−1^ cm^−1^) was measured by the method of Jana and Choudhuri (1981) and the intensity of the yellow color in the supernatant was measured at 410 nm. The MDA content was determined in 50% flowering stage in plants and it indicate level of lipid peroxidation, which was determined as MDA content (molar extinction coefficient at 155 mM^−1^ cm^−1^), according to the method of Heath and Packer (1968). The absorbance was recorded at 532 nm and corrected for non-specific turbidity at 600 nm wavelength. The measured unit of MDA content was represented as μmol g^−1^ fresh weight.

### Antioxidative Enzymes Activities

The enzyme units (EU is per mg protein; specific activity) were calculated as a change in absorbance mg^−1^ protein. The enzyme activities were calculated as EU mg^−1^ protein min^−1^. The enzyme PPO was assayed according to Kar and Mishra (1976) at 420 nm in a spectrophotometer at 50% flowering. The activity of the POX enzyme was measured at 50% flowering by using the method of Kar and Mishra (1976) at 420 nm. The activity of the SOD enzyme was measured at 50% flowering to study variations according to the method of Dhindsa et al. (1981) and the absorbance was recorded at 560 nm. The activity of enzyme CAT was recorded at 50% flowering. The enzyme was assayed according to the method of Aebi (1983). Changes in absorbance at 240 nm at a span of 15 s for 2 min were noted. The enzyme activity was calculated as per gram FW and estimated using extinction coefficient 43.6 for H_2_O_2_ decomposition. It was also estimated on a per mg protein basis and was expressed according to the formula.

EU mg^−1^ protein = δ *A* 240/min × 1000/43.6 × mg protein ml^−1^ reaction mixture.

### Analysis of SS, AA, and PC

The SS content was determined in fully expanded leaf at 50% flowering stage in different treatment levels by anthrone method (glucose standard: 0.1 mg ml^−1^ of distilled water) (Dubois et al., 1956). The sugar content was represented as mg glucose g^−1^ FW by taking the absorbance reading optical density (OD) at 620 nm. Total free AA were determined in fully expanded leaf at the 50% flowering stage in different treatment levels by Ninhydrin reagent (OD at 570 nm) (Yemm et al., 1955). The PC was determined in fully expanded leaf at 50% flowering stage by the method of Bates et al. (1973). A standard curve was prepared using the known concentration of L-Proline (0.1 mg ml^−1^) and contents in sample aliquots were determined by taking an absorbance at 520 nm. The SC was determined in fully expanded leaf at 50% flowering stage in different treatment levels by anthrone method (Dubois et al., 1956). The quantity of starch was recorded by a standard curve as similar to sugar estimation and expressed in terms of glucose. The starch quantity was determined by multiplying the value of glucose concentration with the test extract by a factor of 0.9.

### The FAs Concentration (%)

The FAs in the seeds sample were estimated by gas chromatography. The analysis was carried out at ICAR-Directorate of Rapeseed and Mustard Research, Bharatpur, and Rajasthan, India. Sample containing 10–20 seeds of each genotype under different sowing dates were crushed and kept in hexane (500 μl) overnight. The hexane was transferred in different test-tubes the next day and added with 500 μl sodium methoxide (Sodium methoxide: 80-mg NaOH + 100 ml methanol). After 45 min of incubation, 750 μl sodium chloride was added (8-g NaCl + 100 ml distilled water). The sample was ready and 1 μl of it is injected into the Gas chromatography (GC) channel for analysis.

### Statistical Analysis

The data obtained in the presented experiment was reported as Mean ± Standard deviation (SD), and Microsoft Excel 2010 was used for the analysis. The standard error of mean (±SEM) and critical differences (CD) in between the varieties, date of sowing, and for their interaction were performed by using OPSTAT software developed by O.P. Sheoran, Chaudhary Charan Singh, Haryana Agricultural University, Hisar, Haryana, India. The significant difference in different date of sowing and different varieties with 95% confidence were calculated by SPSS 19.0 (Statistical analysis software; IBM, New York, USA) using Tukey's honestly significant difference (HSD) test. The principal component analysis (PCA) and correlation analysis of different attributes was performed using the R software (V 4.0.2) developed by R core software.

## Results

**Figure 1:**
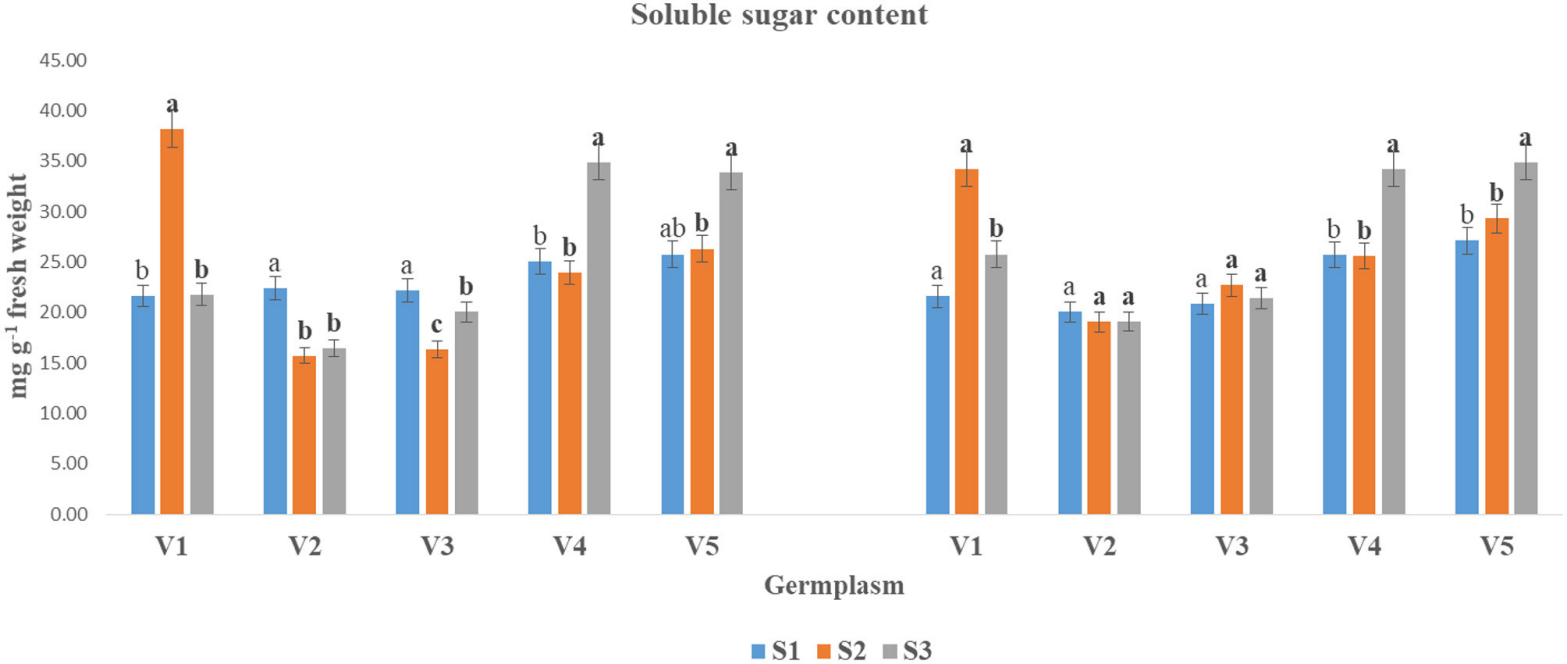
The SS content (mg g^−1^ fresh weight) at mid-flowering stage in the top leaf of mustard varieties sown at three different dates during *rabi* 2017–2018 and 2018–2019. Different date of sowing represented as S1 timely sown (30 October), S2 late sown (15 November) and S3 very late sown (30 November); Varieties symbolize with: V1, PM 25; V2, PM 26; V3, BPR-541-4; V4, RH-406; and V5, Urvashi, respectively. The mean value ± *p* ≤ 0.05 error bar data were presented in the figure and different alphabetical letters indicate the significant difference for each variety under different date of sowing (*p* ≤ 0.05).

**Figure 2:**
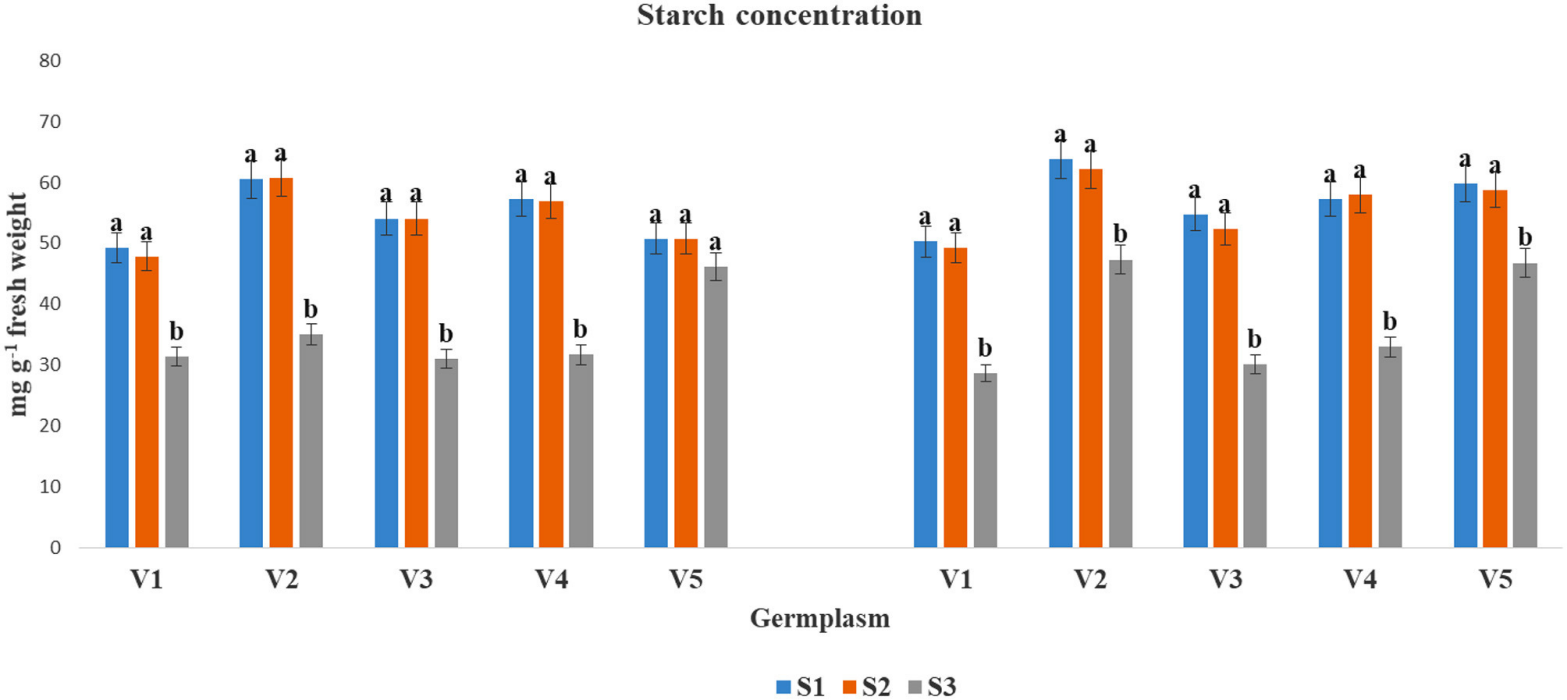
Starch concentrations (mg g^−1^ fresh weight) at mid-flowering stage in top leaf of mustard varieties sown at three different dates during *rabi* 2017–2018 and 2018–2019. Different date of sowing represented as S1 timely sown (30 October), S2 late sown (15 November) and S3 very late sown (30 November 30 Varieties symbolize with: V1, PM 25; V2, PM 26; V3, BPR-541-4; V4, RH-406; and V5, Urvashi, respectively. The mean value ± *p* ≤ 0.05 error bar data were presented in the figure and different alphabetical letters indicate the significant difference for each variety under different date of sowing (*p* ≤ 0.05).

**Figure 3:**
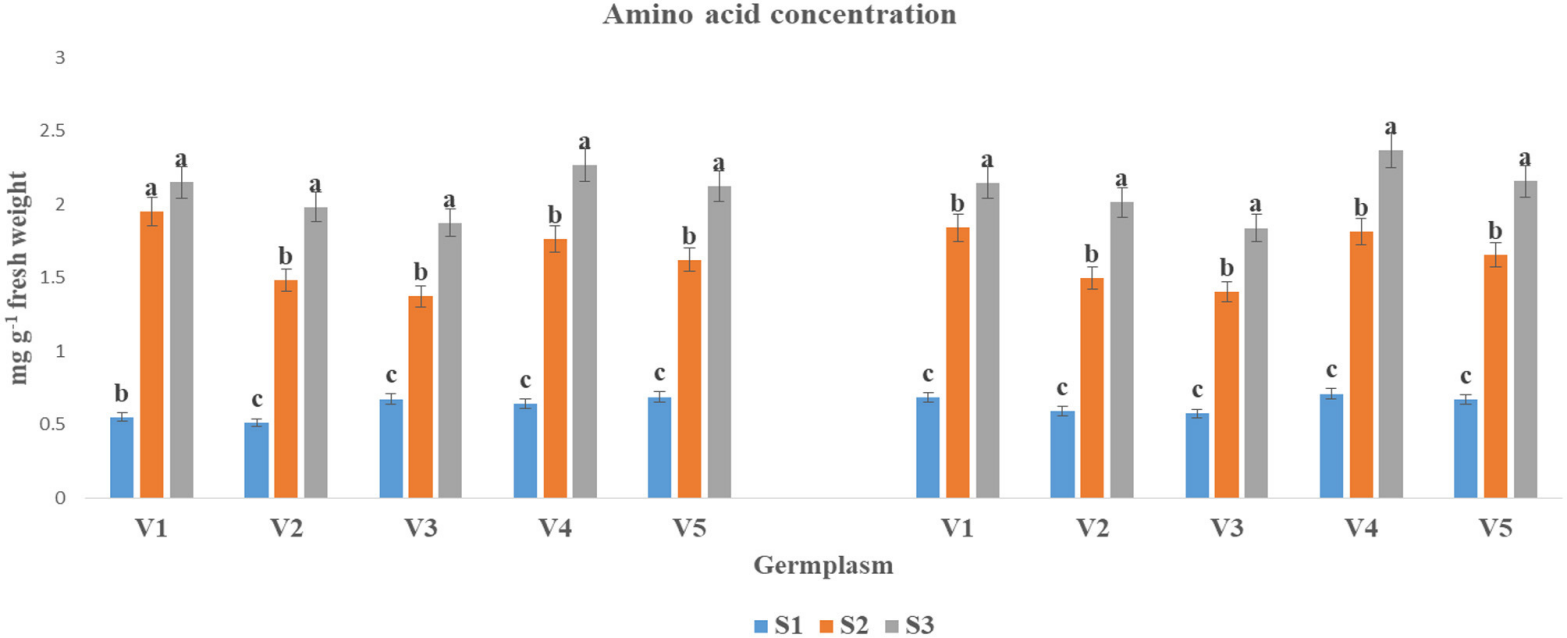
The AA concentration (mg g^−1^ fresh weight) at mid-flowering stage in top leaf of mustard varieties sown at three different dates during *rabi* 2017–2018 and 2018–2019. Different date of sowing represented as S1 timely sown (30 October), S2 late sown (15 November), and S3 very late sown (30 November); Varieties symbolize with: V1, PM 25; V2, PM 26; V3, BPR-541-4; V4, RH-406; and V5, Urvashi, respectively. The mean value ± *p* ≤ 0.05 error bar data were presented in the figure and different alphabetical letters indicate the significant difference for each variety under different date of sowing (*p* ≤ 0.05).

**Figure 4:**
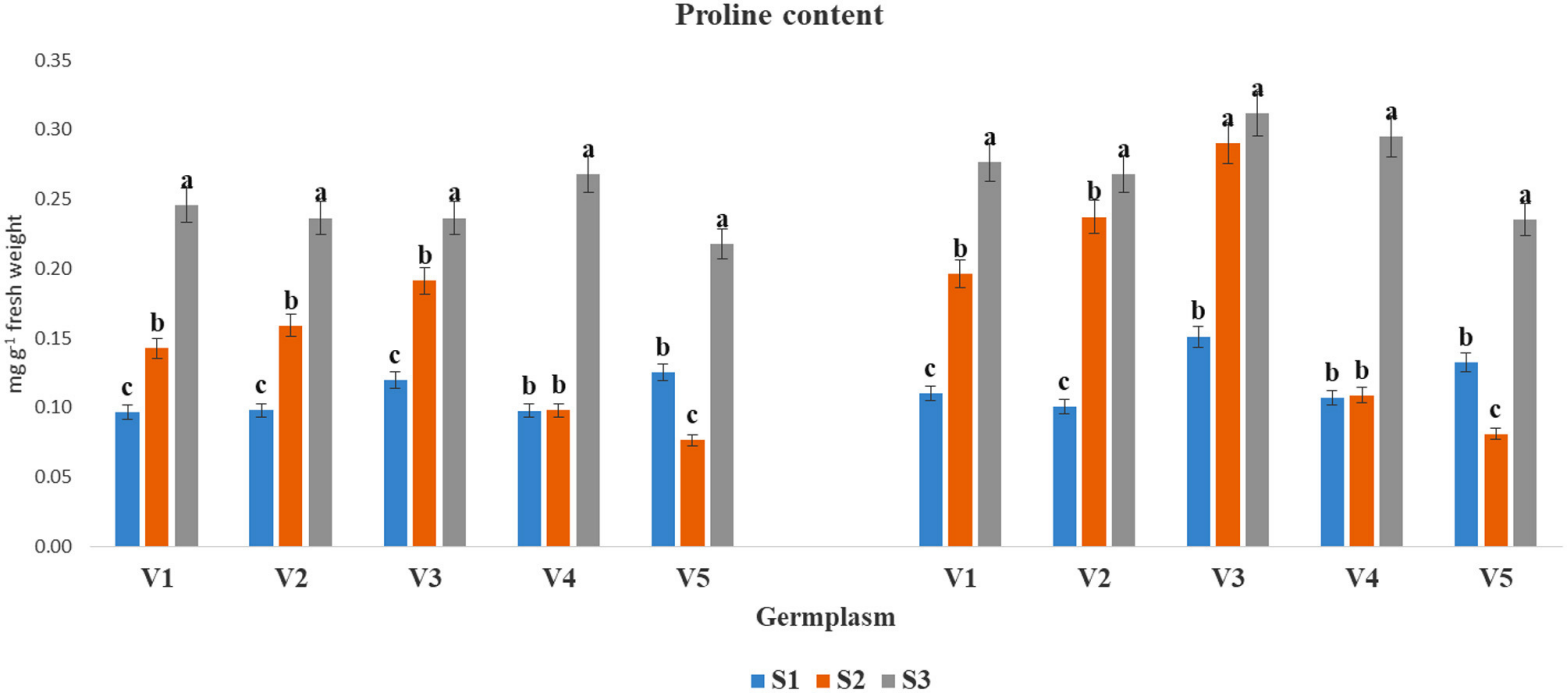
The PC (mg g^−1^ fresh weight) at mid-flowering stage in the top leaf of mustard varieties sown at three different dates during *rabi* 2017–2018 and 2018–2019. Different date of sowing represented as S1 timely sown (30 October), S2 late sown (15 November) and S3 very late sown (30 November); Varieties symbolize with: V1, PM 25; V2, PM 26; V3, BPR-541-4; V4, RH-406; and V5, Urvashi, respectively. The mean value ± *p* ≤ 0.05 error bar data were presented in the figure and different alphabetical letters indicate the significant difference for each variety under different date of sowing (*p* ≤ 0.05).

**Figure 5:**
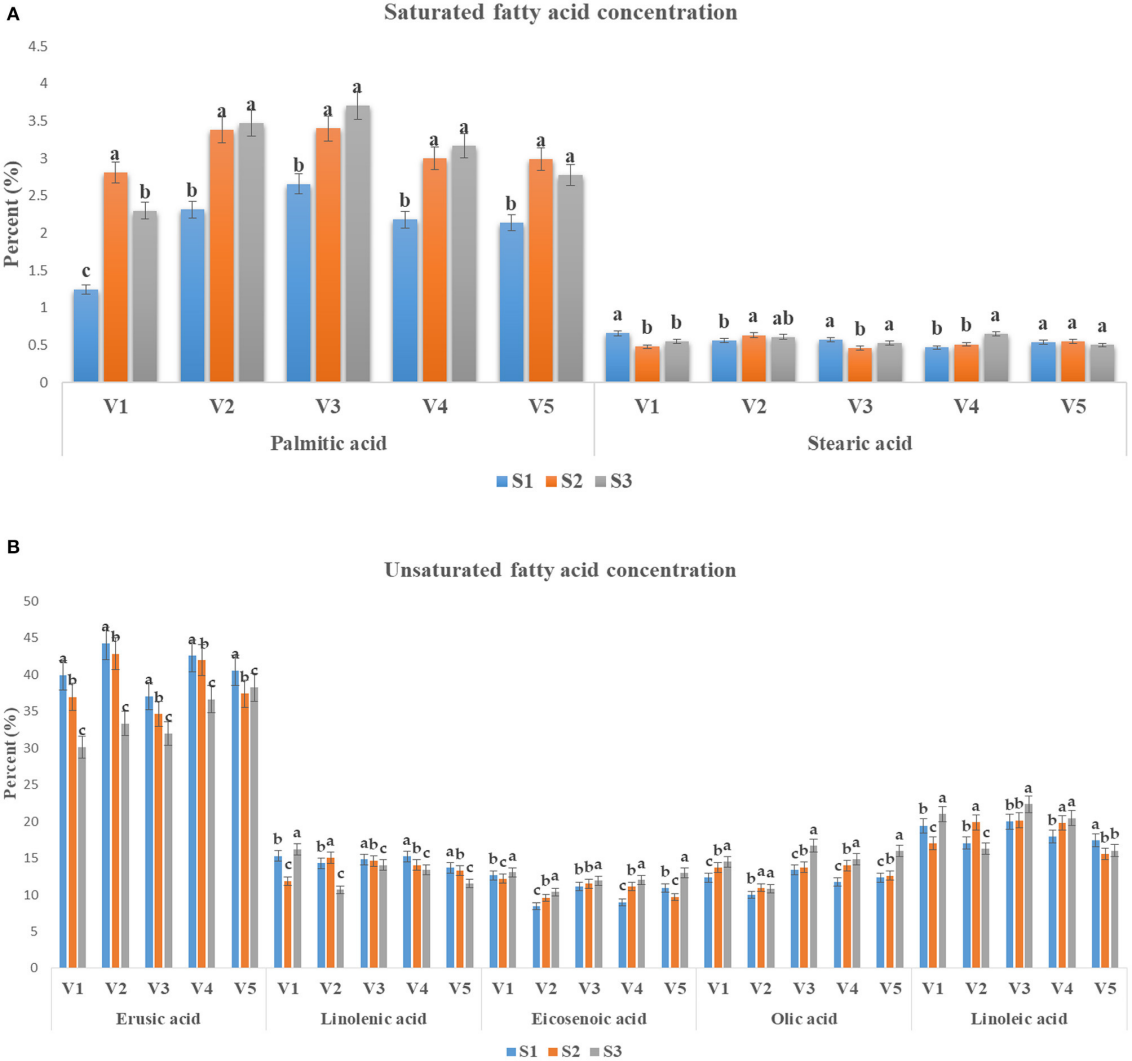
The FA content (%) in mustard varieties sown in three dates during *rabi* 2017–2018 and 2018–2019. **(A,B)** represents the saturated and unsaturated FA content (%), respectively. The graph represents the mean value ± *p* ≤ 0.05 error bar. The different colors symbolized the different FA contents under normal (S1), late (S2), and very late (S3) sowing conditions. Different alphabetical letters in same column indicates the significant difference for each variety under different sowing conditions (*p* ≤ 0.05). Genotype: V1, PM 25; V2, PM 26; V3, BPR-541-4; V4; RH-406; and V5, Urvashi.

The regulation of oxidative stress indicators, enzyme activity, and osmolytes were studied in different mustard germplasms in response to varying sowing times. The effect of treatments and their interactions with genotypes on of the parameters studied at three growth stages including S1 (0 DAT) (day of timely sown), S2 (15 DAT), and S3 (30 DAT). The genotype variations were found at different growth stages of plants in terms of stress indicators, enzyme activity, SS, starch, AA, and FAs (**Tables 1**–**6**; **Figures 1**–**4**, **5A,B**). The data reveals that all these attributes were differentially regulated in mustard genotypes in response to two consecutive cultivation years (2017–2018, 2018–2019). In the following sections, we provided the results associated with the influences of plant sowing time and genotypic variations, and their interactions as well as influences in different physiological attributes in mustard.

### Regulation of Oxidative Stress Indicators

The accumulation of MDA content is an indicator of lipid peroxidation, which generally occurred due to excess level of ROS (hydrogen peroxide, singlet oxygen and superoxide radicle). The resultant effect of ROS induces cellular injury and oxidative damages in plants. In this study, The H_2_O_2_ content (μmol g^−1^ FW) significantly increased in under S3 condition as compared to S1 and S2 in both years during 2017–2018 (Table 1). This trend was followed similarly in both years and all tested germplasms. The increment of H_2_O_2_ content under the S3 condition varied statistically significantly among the tested germplasms. The highest H_2_O_2_ content under the S3 condition was found in V2 (15.89 μmol g^−1^ FW) and in V2 (11.89 μmol g^−1^ FW) during 2017–2018 and 2018–2019, respectively. In contrast, the lowest H_2_O_2_ content was obtained under S3 condition was recorded in V4 (8.26 and 7.26 μmol g^−1^ FW) in both tested years. The MDA content (μmol g^−1^ FW) was significantly increased under the S3 condition as compared to S1 and S2 in both tested years, whereas it found to be reduced in S3 sowing (Table 2). The increment of MDA content under the S3 condition varied statistically significantly among the tested germplasms. The highest MDA content under S3 condition was found in genotype V1 for 2.54 and 2.47 μmol g^−1^ FW, and the lowest MDA content under S3 condition was recorded in genotype V4 for 0.68 and 0.75 μmol g^−1^ FW, respectively, in both tested years.

**Table 1:** Hydrogen peroxide content (μmol g^−1^ fresh weight) at mid-flowering stage in top leaf of mustard varieties sown at three different dates during *rabi* 2017–2018 and 2018–2019.

**Genotypes**	**2017–2018**			**Mean**	**2018–2019**			**Mean**
	**S1**	**S2**	**S3**		**S1**	**S2**	**S3**	
**V1**	5.51 ± 0.76b	6.62 ± 0.91b	11.64 ± 1.89a	**7.92**	5.05 ± 0.43c	6.28 ± 0.70b	9.31 ± 0.23a	**6.88**
**V2**	7.21 ± 0.63b	8.66 ± 0.76b	15.89 ± 2.79a	**10.59**	6.88 ± 0.56b	7.66 ± 0.39b	11.89 ± 0.17a	**8.81**
**V3**	5.67 ± 0.54c	6.80 ± 0.65b	12.02 ± 0.27a	**8.16**	5.33 ± 0.10c	6.49 ± 0.22b	10.36 ± 0.39a	**7.39**
**V4**	4.16 ± 0.88a	4.99 ± 1.05a	8.26 ± 0.62a	**5.80**	3.83 ± 0.44b	4.66 ± 0.21b	7.26 ± 0.62a	**5.25**
**V5**	4.39 ± 0.67b	5.26 ± 0.81b	8.82 ± 1.68a	**6.16**	4.09 ± 0.33b	4.95 ± 0.44b	8.16 ± 0.83a	**5.73**
**Mean**	**5.39**	**6.47**	**11.33**		**5.03**	**6.01**	**9.39**	
		**±SEM**		**CD (5%)**		**SEM±**	**CD (5%)**	
Sowing date (S)		0.33		0.96		0.15	0.44	
Genotype (G)		0.43		1.24		0.20	0.57	
S × G		0.74		2.15		0.34	0.99	

*# Different date of sowing represented as S1 timely sown (30 October), S2 late sown (15 November) and S3 very late sown (30 November); Varieties symbolize with: V1, PM 25; V2, PM 26; V3, BPR-541-4; V4, RH-406; and V5, Urvashi, respectively. The mean ± SD data were presented in table and different alphabetical letters in the same row indicate the significant difference for each variety under different date of sowing (p ≤ 0.05); CD at 5%, and Mean ± SEM, data between sowing dates (S), genotype (G), and S × G interactions were also highlighted*.

**Table 2:** Malondialdehyde (MDA) content (μmol g^−1^ fresh weight) at mid-flowering stage in the top leaf of mustard varieties sown at three different dates during *rabi* 2017–2018 and 2018–2019.

**Genotypes**	**2017–2018**			**Mean**	**2018–2019**			**Mean**
	**S1**	**S2**	**S3**		**S1**	**S2**	**S3**	
**V1**	0.93 ± 0.02b	1.11 ± 0.12b	2.54 ± 0.09a	**1.52**	1.32 ± 0.01b	1.22 ± 0.05b	2.47 ± 0.03a	**1.67**
**V2**	2.30 ± 0.01a	2.36 ± 0.01a	1.63 ± 0.04b	**2.10**	1.80 ± 0.01b	2.27 ± 0.02a	1.71 ± 0.04c	**1.93**
**V3**	1.26 ± 0.01a	0.89 ± 0.01b	0.93 ± 0.08b	**1.02**	0.74 ± 0.01c	0.97 ± 0.01a	0.86 ± 0.03b	**0.86**
**V4**	0.42 ± 0.01a	0.67 ± 0.05a	0.68 ± 0.17a	**0.59**	0.36 ± 0.02b	0.77 ± 0.03a	0.75 ± 0.19a	**0.62**
**V5**	1.03 ± 0.03c	1.47 ± 0.01b	1.57 ± 0.04a	**1.35**	0.84 ± 0.03c	1.47 ± 0.01b	1.65 ± 0.03a	**1.32**
**Mean**	**1.19**	**1.30**	**1.47**		**1.01**	**1.34**	**1.49**	
		**SEM±**		**CD (5%)**		**SEM±**	**CD (5%)**	
Sowing date (S)		0.02		0.06		0.02	0.05	
Genotype (G)		0.03		0.08		0.02	0.06	
S × G		0.05		0.13		0.04	0.11	

*#Different date of sowing represented as S1 timely sown (30 October), S2 late sown (15 November) and S3 very late sown (30 November); Varieties symbolize with: V1, PM 25; V2, PM 26; V3, BPR-541-4; V4, RH-406; and V5, Urvashi, respectively. The mean ± SD data were presented in table and different alphabetical letters in the same row indicate the significant difference for each variety under different date of sowing (p ≤ 0.05); CD at 5%, and Mean ± SEM, data between sowing dates (S), genotype (G), and S × G interactions were also highlighted*.

### Antioxidant Enzymes Activity

The PPO activity (EU mg^−1^ protein min^−1^) was significantly increased under the S3 condition as compared to S1 and S2 in both years (Table 3). However, the PPO activity was lowest in S2 condition as compared to S1 and S3. The increment of PPO activity under the S3 condition varied statistically significant among the tested germplasms. The highest PPO activity under the S3 condition was found in V5 (0.048 and 0.049 EU mg^−1^ protein min^−1^) in both tested years and the lowest PPO activity under S3 condition was recorded in V3 (0.037 EU mg^−1^ protein min^−1^).

**Table 3:** Polyphenol oxidase (PPO) activity (EU mg^−1^ protein min^−1^) at mid-flowering stage in top leaf of mustard varieties sown at three different dates during *rabi* 2017–2018 and 2018–2019.

**Genotypes**	**2017–2018**			**Mean**	**2018–2019**			**Mean**
	**S1**	**S2**	**S3**		**S1**	**S2**	**S3**	
**V1**	0.046 ± 0.003a	0.018 ± 0.002b	0.046 ± 0.001a	**0.037**	0.050 ± 0.003a	0.018 ± 0.002b	0.047 ± 0.001a	**0.038**
**V2**	0.035 ± 0.002a	0.018 ± 0.002b	0.038 ± 0.004a	**0.030**	0.035 ± 0.002a	0.018 ± 0.002b	0.041 ± 0.00a	**0.031**
**V3**	0.027 ± 0.005b	0.018 ± 0.002c	0.037 ± 0.001a	**0.027**	0.027 ± 0.005b	0.018 ± 0.002b	0.037 ± 0.001a	**0.027**
**V4**	0.037 ± 0.002b	0.026 ± 0.00c	0.045 ± 0.002a	**0.036**	0.038 ± 0.002a	0.026 ± 0.00b	0.046 ± 0.002a	**0.037**
**V5**	0.041 ± 0.001a	0.027 ± 0.003b	0.048 ± 0.002a	**0.039**	0.043 ± 0.001b	0.025 ± 0.001c	0.049 ± 0.001a	**0.039**
**Mean**	**0.037**	**0.022**	**0.043**		**0.039**	**0.021**	**0.044**	
		**SEM±**		**CD (5%)**		**SEM±**	**CD (5%)**	
Sowing date (S)		0.0008		0.002		0.0008	0.002	
Genotype (G)		0.0010		0.003		0.0006	0.002	
S × G		0.0017		0.005		0.0013	0.004	

*#Different date of sowing represented as S1 timely sown (30 October), S2 late sown (15 November) and S3 very late sown (30 November); Varieties symbolize with: V1, PM 25; V2, PM 26; V3, BPR-541-4; V4, RH-406; and V5, Urvashi, respectively. The mean ± SD data were presented in table and different alphabetical letters in the same row indicate the significant difference for each variety under different date of sowing (p ≤ 0.05); CD at 5%, and Mean ± SEM, data between sowing dates (S), genotype (G), and S × G interactions were also highlighted*.

The POX was non-significant under S3 condition as compared to S1 and S2 in both years, except in V3 where it increased under the S3 sowing (Table 4). The highest POX activity under S3 condition was found in V5 for 2.02 and 1.98) then genotype V4 exhibited 2.00 and 1.99, whereas, the lowest POX activity under the S3 condition was recorded in V2 (1.70) and V3 (1.72), respectively.

**Table 4:** The POX activity (EU mg^−1^ protein min^−1^) at mid-flowering stage in top leaf of mustard varieties sown at three different dates during *rabi* 2017–2018 and 2018–2019.

**Genotypes**	**2017–2018**			**Mean**	**2018–2019**			**Mean**
	**S1**	**S2**	**S3**		**S1**	**S2**	**S3**	
**V1**	1.96 ± 0.20a	1.78 ± 0.08a	1.95 ± 0.05a	**1.89**	1.89 ± 0.17a	1.81 ± 0.05a	1.92 ± 0.05a	**1.87**
**V2**	1.61 ± 0.11a	1.54 ± 0.05a	1.70 ± 0.08ae	**1.62**	1.65 ± 0.09a	1.70 ± 0.11a	1.74 ± 0.04a	**1.70**
**V3**	1.16 ± 0.01b	1.65 ± 0.10a	1.78 ± 0.18a	**1.53**	1.19 ± 0.03b	1.64 ± 0.07a	1.72 ± 0.15a	**1.52**
**V4**	1.90 ± 0.11a	1.77 ± 0.07a	2.00 ± 0.15a	**1.89**	1.87 ± 0.14ba	1.74 ± 0.04a	1.99 ± 0.16a	**1.87**
**V5**	1.89 ± 0.05a	2.06 ± 0.07a	2.02 ± 0.08a	**1.99**	1.92 ± 0.06a	2.09 ± 0.08a	1.98 ± 0.04a	**2.00**
**Mean**	**1.70**	**1.76**	**1.89**		**1.70**	**1.80**	**1.87**	
		**SEM±**		**CD (5%)**		**SEM±**	**CD (5%)**	
Sowing date (S)		0.03		0.09		0.04	0.11	
Genotype (G)		0.04		0.12		0.03	0.08	
S × G		0.07		0.21		0.06	0.18	

*#Different date of sowing represented as S1 timely sown (30 October0), S2 late sown (15 November) and S3 very late sown (30 November 30); Varieties symbolize with: V1, PM 25; V2, PM 26; V3, BPR-541-4; V4, RH-406; and V5, Urvashi, respectively. The mean ± SD data were presented in table and different alphabetical letters in the same row indicate the significant difference for each variety under different date of sowing (p ≤ 0.05); CD at 5%, and Mean ± SEM, data between sowing dates (S), genotype (G), and S × G interactions were also highlighted*.

The SOD activity (EU mg^−1^ protein min^−1^) was elevated at S3 condition as compared to S1 and S2 in both years (Table 5). The increment of SOD activity under the S3 condition varied statistically significantly among the tested genotypes. The highest SOD activity under S3 condition was found in V3 (11.56 and 11.41) and followed by V1 (10.61 and 10.84) in both tested years. In contrast, the lowest SOD activity under S3 condition was recorded in V4 (7.84) and V5 (7.93), respectively, in two cultivation years.

**Table 5:** The SOD activity (EU mg^−1^ protein min^−1^) at mid-flowering stage in the top leaf of mustard varieties sown at three different dates during *rabi* 2017–2018 and 2018–2019.

**Genotypes**	**2017–2018**			**Mean**	**2018–2019**			**Mean**
	**S1**	**S2**	**S3**		**S1**	**S2**	**S3**	
**V1**	4.76 ± 0.18c	5.29 ± 0.30b	10.61 ± 0.48a	**6.88**	4.87 ± 0.10c	5.62 ± 0.26b	10.84 ± 0.26a	**7.11**
**V2**	4.55 ± 0.54b	5.06 ± 0.60b	10.50 ± 0.99a	**6.70**	4.45 ± 0.40c	5.39 ± 0.55b	10.23 ± 0.75a	**6.69**
**V3**	5.21 ± 0.20b	5.79 ± 0.30b	11.56 ± 0.56a	**7.52**	5.11 ± 0.11c	5.95 ± 0.08b	11.41 ± 0.33a	**7.49**
**V4**	3.45 ± 0.22c	3.83 ± 0.24b	7.84 ± 0.46a	**5.04**	3.61 ± 0.14b	4.00 ± 0.12b	7.97 ± 0.35a	**5.19**
**V5**	3.49 ± 0.20b	3.88 ± 0.39b	7.93 ± 0.52a	**5.10**	3.82 ± 0.68b	4.28 ± 0.42b	7.93 ± 0.52a	**5.34**
**Mean**	**4.29**	**4.77**	**9.69**		**4.37**	**5.05**	**9.68**	
		**SEM±**		**CD (5%)**		**SEM±**	**CD (5%)**	
Sowing date (S)		0.14		0.41		0.12	0.34	
Genotype (G)		0.18		0.53		0.15	0.44	
S × G		0.32		0.92		0.26	0.76	

*#Different date of sowing represented as S1 timely sown (30 October), S2 late sown (15 November) and S3 very late sown (30 November); Varieties symbolize with: V1, PM 25; V2, PM 26; V3, BPR-541-4; V4, RH-406; and V5, Urvashi, respectively. The mean ± SD data were presented in table and different alphabetical letters in the same row indicate the significant difference for each variety under different date of sowing (p ≤ 0.05); CD at 5%, and Mean ± SEM, data between sowing dates (S), genotype (G) and S × G interactions were also highlighted*.

The CAT activity (EU mg^−1^ protein min^−1^) was tested in all the germplasm and significantly increased under S3 condition as compared to S1 and S2 in both years (Table 6). Although the lowest CAT activity was recorded in S2 as compared to S1 and S3. This trend was followed similarly in both years and in all the tested germplasms. The increment of CAT activity under the S3 condition varied statistically significantly among the tested germplasms. The highest CAT activity under S3 condition was found in V5 (22.71 and 24.71 EU mg^−1^ protein min^−1^) and followed by V1 (21.05 and 23.31 EU mg^−1^ protein min^−1^) in both tested years. Whereas, the lowest CAT activity under S3 condition was recorded in V2 (18.37 and 17.04 EU mg^−1^ protein min^−1^) in the tested years.

**Table 6:** The CAT activity (EU mg^−1^ protein min^−1^) at mid-flowering stage in top leaf of mustard varieties sown at three different dates during *rabi* 2017–2018 and 2018–2019.

**Genotypes**	**2017–2018**			**Mean**	**2018–2019**			**Mean**
	**S1**	**S2**	**S3**		**S1**	**S2**	**S3**	
**V1**	19.51 ± 0.66a	15.02 ± 0.82b	21.05 ± 0.00a	**18.53**	18.85 ± 1.13a	17.02 ± 0.82b	23.31 ± 1.04a	**19.73**
**V2**	17.37 ± 0.47a	13.68 ± 1.25b	18.37 ± 1.24a	**16.48**	16.71 ± 0.47a	13.02 ± 0.82b	17.04 ± 0.82a	**15.59**
**V3**	18.37 ± 0.47a	11.68 ± 1.25b	19.04 ± 0.82a	**16.36**	16.70 ± 0.47a	11.02 ± 1.42b	18.70 ± 1.25a	**15.48**
**V4**	19.75 ± 0.67a	12.36 ± 1.25b	20.71 ± 0.47a	**17.61**	18.75 ± 1.75a	12.36 ± 1.25b	21.71 ± 1.89a	**17.61**
**V5**	19.38 ± 0.99b	14.69 ± 0.47c	22.71 ± 1.25a	**18.93**	18.38 ± 0.94b	16.86 ± 1.25b	24.71 ± 0.94a	**19.99**
**Mean**	**18.88**	**13.49**	**20.38**		**17.88**	**14.05**	**21.10**	
		**SEM±**		**CD (5%)**		**SEM±**	**CD (5%)**	
Sowing date (S)		0.42		1.22		0.35	1.02	
Genotype (G)		0.54		1.57		0.45	1.32	
S × G		0.94		2.72		0.79	2.28	

*#Different date of sowing represented as S1 timely sown (30 October), S2 late sown (15 November) and S3 very late sown (30 November); Varieties symbolize with: V1, PM 25; V2, PM 26; V3, BPR-541-4; V4, RH-406; and V5, Urvashi, respectively. The mean ± SD data were presented in table and different alphabetical letters in the same row indicate the significant difference for each variety under different date of sowing (p ≤ 0.05); CD at 5%, and Mean ± SEM data between sowing dates (S), genotype (G), and S × G interactions were also highlighted*.

### The SS, Starch, Free AA, and Proline Contents

The SS, starch, free AA, and PC (mg g^−1^ FW) at the S2 stage was determined in the first fully expanded leaf from the top in plants under different sowing dates (S) during 2017–2018 and 2018–2019, respectively. The differences in the discussed biochemical parameters were significant with respect to sowing date (S) genotype (G), and interaction S × G.

In this study, The SS content was significantly declined under the S3 condition as compared to the S1 and S2 stages in both years (Figure 1). The reduction in SS content under the S3 condition varied significantly among the tested genotypes. Although the SS content was non-significant in germplasms V2 and V3 in the year 2018–2019 in three respective to sowing stages. Germplasm V3 and V4 showed the inverse relationship and increased in S3 condition as compared to S1 and S2. Germplasm V4 (34.91 and 34.24) and V5 (33.87 and 34.84) showed the highest SS content in both years at S3 conditions. While the lowest SS contents were found in V2 (16.40 and 19.07 mg g^−1^ FW) followed by V3 in both years, respectively.

The SC accumulation was found to be reduced under the S3 condition as compared to S1 and S2 in both years, except in V5 at 2017–2018, where the data were non-significant (Figure 2). However, the reduction of SC under S3 condition varied significantly among the tested germplasms. The highest SC content under at S3 condition was found for V5 (46.12 and 46.79) followed by V2 (34.99 and 47.32) in both the cultivation years. The lowest SC contents under the S3 condition was recorded in V1 (31.35 and 28.68) followed by V3 (31.07) during 2017–2018, and V1 (28.68) during 2018–2019.

The AA concentration was significantly lifted at S3 condition as compared to S1 and S2 in both years (Figure 3). The highest AA concentration under S3 condition was found in V4 (2.27 and 2.37) and V1 (2.15 and 2.15). In contrast, the lowest AA concentration was observed at S3 condition for V3 (1.87 and 1.84).

The accumulation of PC (mg g^−1^ FW) was significantly increased at S3 condition as compared to S1 and S2 in both years (Figure 4). The highest PC under S3 condition was found in V4 (0.27) and in V3 (0.31) during 2017–2018 and 2018–2019, respectively, whereas, the lowest proline accumulation was obtained under the S3 condition for V5 (0.22 and 0.24) in two cultivated years, respectively.

### The FAs Contents (%)

Major FAs including oleic acid, linolenic acid, linoleic acid and erucic acids were measured at three different growing stages of five distinct genotypes. The palmitic acid showed maximum level in V2 and V3 genotypes, respectively. Although no significant differences have been found for palmitic as at stages S2 and S3, respectively (Figure 5A). However, no substantial variation has been found for stearic acid accumulation among the V1 V2 and V5 genotypes in S2 and S3 growth stages (Figure 5A). A considerable increased level of erucic acid content was found inV1, V2, V4, and V5, respectively specially at S1 and S2 growth stages of mustard (Figure 5B).

### Correlation and PCA

**Figure 6:**
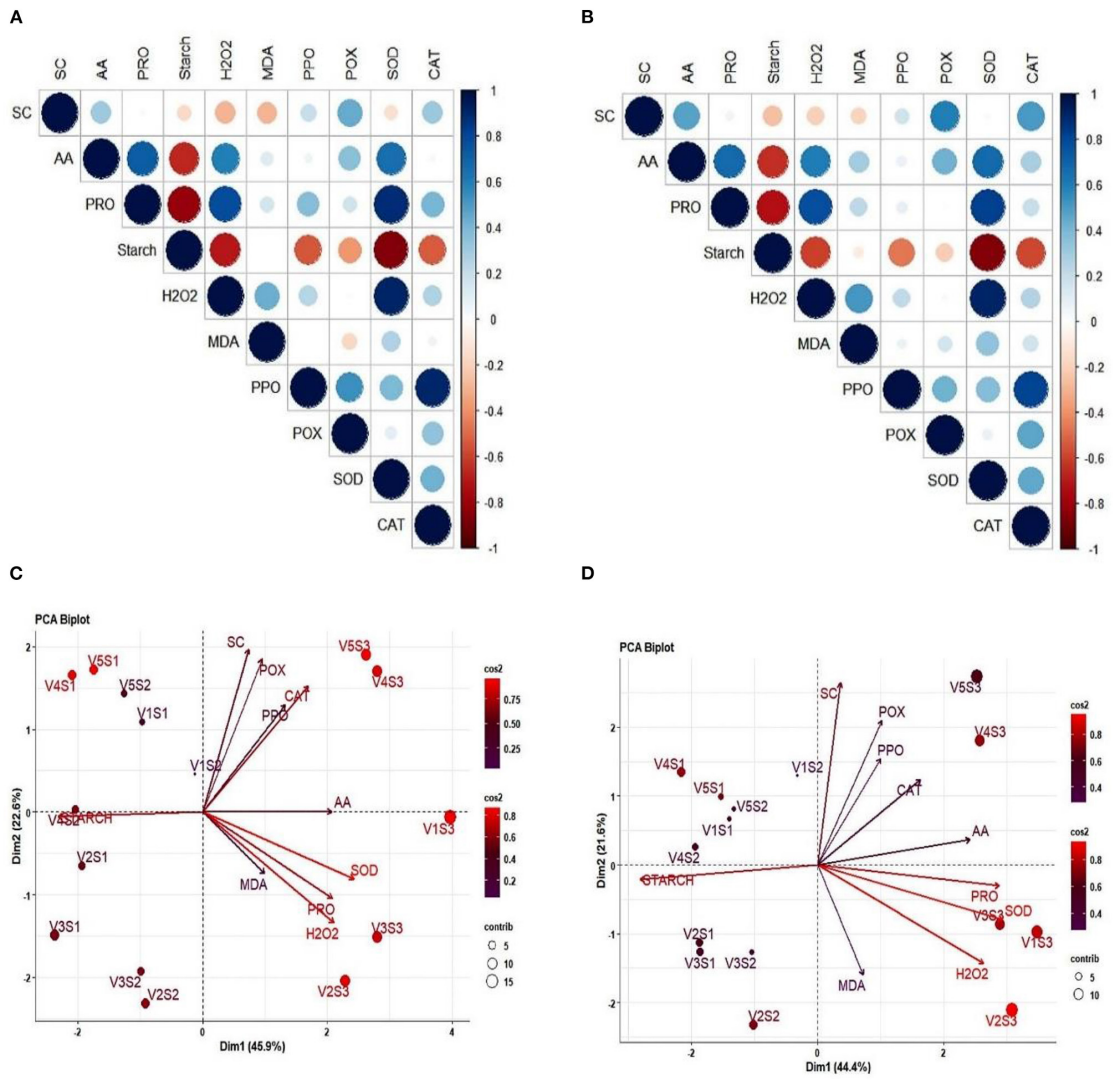
Correlations 6 **(A,B)** and PCA 6 **(C,D)** for different biochemical traits observed in mustard genotypes sown in three different dates during 2017–2018 and 2018–2019. Panels **(A,B)** represents the correlation matrix using correlogram and the different colors shows the correlation between variable (blue color showed positive, while red negative) and color intensity shows the correlation coefficient matrix (dark and big circle showed the strong correlation). Panels **(C,D)** PCA analysis shows the principal component varied under different sowing condition and the variation values are highlighted on –*x* and –*y*-axis. V1, PM 25; V2, PM 26; V3, BPR-541-4; V4, RH-406; and V5, Urvashi. S1 timely sown (30 October), S2 late sown (15 November), and S3 very late sown (30 November), respectively. Superoxide dismutase activity (SOD), hydrogen peroxide (H_2_O_2_), proline content (Pro), peroxidase (POX) activity, sugar content (SC), and catalase (CAT) activity, polyphenol oxidase (PPO), and malondialdehyde (MDA) content.

A positive correlation was found among the AA, proline, MDA, H_2_O_2_ contents, POX, SOD, and CAT activities in 2017–2018, and 2018–2019, respectively. However, AA and proline content; PC and hydrogen peroxide, SOD; hydrogen peroxide and SOD activity; PPO activity and CAT activity exhibited strong and positive correlation. A significant and negative association was observed between AA and starch content; proline and starch content; starch and SOD activity (Figures 6A,B). The PCA of the five mustard varieties sown in three different dates has together accounted for 66.0 and 68.5% of the total variation during Rabi season of 2017–2018 and 2018–2019, respectively (Figures 6C,D). In 2017–2018, PC1 explained 44.4%, while PC2 explained 21.6% of the total variation whereas, in 2018–2019, PC1 explained 45.9% and PC2 explained 22.5% of the total variation with all the characters being positively loaded.

## Discussion

In this investigation level of one of the ROS that is H_2_O_2_, was quantified. As compared to 30 October and 15 November sowings, the H_2_O_2_ content increased significantly in 30 November sown crops (Table 1). The MDA content was also significantly higher on 30 November sown crops. These observations further proved that as compared to 30 October and 15 November sowings, 30 November sown crop experienced severe stress, and the ROS scavenging system was not so efficient to detoxify the generated ROS causing severe oxidation of membrane lipids and accumulation of MDA. These observations are in accordance with the observation of Rai A. N. et al. (2020) and Ahmed et al. (2019). It has been explained earlier that November 30 sown crop experienced higher oxidative stress, as they exhibited higher membrane damage which was evident from increased H_2_O_2_ (Table 1) and MDA contents (Table 2). Moreover, it has been considered that the tolerant germplasm accumulates less H_2_O_2_ and MDA content because of well-developed defense system such as antioxidant enzymes, osmoprotectants, stress-related genes and transcription factors and crosstalk's signaling strategies (Soengas et al., 2018; Rai K. K. et al., 2020).

The plants with efficient ROS scavenging systems are reported to perform better under abiotic stresses (Katano et al., 2018; Ahmad Z. et al., 2020). Increased activities of SOD, CAT, PPO, and POX in the cells play a significant role in detoxifying ROS under different abiotic stresses (Zhang et al., 2015; Medina et al., 2021; Su et al., 2021). The SOD is an important antioxidant metalloenzyme associated to conversion of harmful O^2−^ to less toxic H_2_O_2_ and O_2_ molecules. The genome wide identification and characterization of *SOD* gene family revealed that *B. juncea* have 29 *SOD* genes and among them 10 of *SOD* genes were linked to abiotic stresses like drought heat, etc. (Verma et al., 2019). Like the SOD enzyme CAT also worked as antioxidant and detoxify ROS through conversion into less harmful H_2_O molecules. Further, the genome wide analysis of CAT gene family revealed that the *B. napus* have 14 *CAT* genes and among them, eight genes related to different abiotic stresses (Raza et al., 2021). In this study-specific activities of SOD, CAT increased significantly, particularly in 30 November sown crop. However, their activities were comparable in 30 October and 15 November sown crops. Like, SOD and CAT, POX also oxidize several ROS using H_2_O_2_ into less toxic molecules and provide tolerance against multiple abiotic and biotic stresses (Balfagón et al., 2018; Rajput et al., 2021). PPO is important metalloproteinase, catalyzes the oxidation of phenols to quinones and further synthesizes the melanin and crosslinked protein polymers. Also, PPO has crucial role in defense against the biotic and abiotic stresses (Zhang and Sun, 2021). The PPO enzyme has important role in repairing of membrane damage caused by the stresses and strengthening of cell membranes (He et al., 2021). Therefore, it is concluded that increased activities of studied ROS scavenging enzymes are crucial for identification of screening of stress tolerant lines.

Increased ROS causes for oxidative stress and affects the cellular mechanisms drastically (Medina et al., 2021). Stress tolerance involves the active accumulation of compatible solutes such as SS, organic acids (primarily AA), and potassium ions, which help in turgor maintenance of the cells (Raza, 2020). Increased PC under elevated temperature has been reported in mustard (Ghasemi et al., 2016). However, increased levels of SS and reduction in SC have been reported in many crop plants (Ahmad M. et al., 2021). In the present investigation, changes in SS (Figure 1), starch (Figure 2), free AA (Figure 3), and proline (Figure 4) contents with variation in sowing dates indicated that change in the sowing date affected these metabolites. With a delay in sowing, SS content in the first fully leaf from top increased, and concomitantly SC decreased. Although these two metabolites did not change much on 30 October and 15 November sown crops, their levels reduced significantly when the crop was sown on November 30. The reduction in SS and starch contents in 30 November sown crops indicated that late sowing reduced photosynthesis significantly. The reduction in photosynthesis under high temperature is documented (Ougham et al., 2008; Qazi et al., 2014), and therefore this might be one of the major reasons for a significant reduction in dry matter production on 30 November sown crop of mustard. The genotypic variations in SS and starch contents indicated that photosynthesis and synthesis as well as a breakdown of starch in different genotypes of mustard respond differently to heat stress. Free AA and proline increased significantly in mustard when sowing was done on 30 November. Observations indicated that these probably constitutes play a noteworthy role in thermotolerance in mustard by playing a role in osmotic adjustment (Siddique et al., 2018). Proline accumulation was relatively higher in BPR 541-4 (V3) and RH-406 (V4). Therefore, it is inferred that the accumulation of proline in response to high-temperature stress may be considered as a screening parameter to identify stress-tolerant lines. As, free AA are precursor of proteins, and secondary metabolites, which have crucial role in osmotic adjustment (Zou et al., 2016).

Under high-temperature, an increase in erucic acid and a decrease in oleic acid contents have been observed in *Brassica hirta*. The seeds of *Brassica* spp. having initially high erucic acid exhibited further increase in erucic acid and decrease in oleic acid contents under low temperature (12–17°C); however, the seeds of low erucic acid-containing *Brassica* spp. exhibited lower oleic acid and higher linolenic acid contents. A reverse had been reported at higher temperatures (Yaniv et al., 1995). Fayyaz-ul-Hassan et al. (2005) reported considerable variations in oleic acid and erucic acid contents with variations in cultivars and sowing dates. They showed that genotype Shiralee contained the highest oil content (41.81%), whereas Zafar-2000 showed the lowest (38.86%). Moreover, Zafar-2000 accumulated the maximum percent of oleic acid (63.77%) and the lowest of erucic acid (21.78%). Turhan et al. (2011) reported considerable changes in FA composition in mustard oil on an account of late sowing. Wilkes et al. (2013) reported lower oleic acid and higher linoleic acid levels in late sown mustard. In the present investigation increase in contents of eicosenoic acid and reduction in the level of erucic acid in seeds of 30 November sown mustard genotypes (Figure 5) indicated that these FAs respond differently to terminal heat stress. Increased in the level of FAs *viz*. palmitic, stearic, oleic, linoleic, and reduction in linolenic acid under the terminal heat stress indicated that the metabolism of these FAs is affected differently under the terminal heat stress. The estimation of different FAs contents in seeds of mustard genotypes sown at different dates are given in Figures 5A,B. As compared to 30 October, sowing palmitic acid, stearic acid, oleic acid, and linoleic acid increased in seeds of 15 November and 30 November sown crops. Linolenic acid content decreased as sowing was delayed. Eicosenoic content increased in seeds when sowing was delayed while and erucic acid content decreased. Further, the transcriptional regulatory molecular mechanism behind the overall improvement of biochemical attributes is also important from sustainability perspective (Higashi and Saito, 2019; Shabbir et al., 2021).

Furthermore, PCA under stress conditions showed the maximum contribution of SOD, hydrogen peroxide, proline content, sugar content, and CAT activity toward total variation whereas, SC contributed the least toward the variation. PCA and correlation studies altogether indicated the strong and positive association among AA, proline content, hydrogen peroxide, SOD activity, and CAT activity which were involved in osmotic adjustment and thereby protecting the integrity of cell membranes and important macromolecules of plants under stress conditions (Figure 6).

## Conclusions

This study explored the mechanisms associated with oxidative indicators, and responses of Indian mustard to varied sowing times, and field temperatures. The results suggested that the late sowing (S3) increased the concentrations of ROS, MDA, H_2_O_2_, and lipid peroxidation, which are responsible for enhanced oxidative stress in the plants. The variety RH-406 (V4) under late sown and elevated temperature exhibited higher SS, AA, PC, SOD, POX, and CAT activities and reduced MDA and hydrogen peroxide content indicating the suitability of this variety for delayed sowing and terminal heat stress conditions. Likewise, the significant differences occurred in the case of saturated (palmitic and stearic acid) and unsaturated (oleic acid, linoleic acid, linolenic acid, eicosenoic acid, and erucic acid) FA contents and variety V4 (RH-406) showed the least fluctuation under delayed sowing; suitable for higher yield and oil quality traits. The overall results suggested that the screening of Indian mustard varieties based on oxidative stress indicators and antioxidants attributes is crucial to identify potential varieties for terminal heat stress and can be used for future stress breeding programs. Thus, from the current study findings, it may be recommended that variety V4 (RH-406) is best for the Uttar Pradesh, East region, India, where the terminal heat stress frequently occurs and it is suggested that farmer should follow timely sowing on first week of November to avoid terminal heat stress.

## Data Availability Statement

The datasets presented in this study can be found in online repositories. The names of the repository/repositories and accession number(s) can be found in the article/Supplementary Material.

## Author Contributions

JC and JS: conceptualization. JC and BD: methodology, investigation, and writing—original draft preparation. JS and RS: software. BD, UM, and HA: validation. MSa and RS: formal analysis. MB: resources. BD: data curation. WS, JS, RS, MR, MZ, MSk, and AE: writing—review and editing. All authors have read and agreed to the published version of the manuscript.

## Funding

The present work was supported by Researchers Supporting Project Number (RSP-2021/390), King Saud University, Riyadh, Saudi Arabia.

## Conflict of Interest

The authors declare that the research was conducted in the absence of any commercial or financial relationships that could be construed as a potential conflict of interest.

## Publisher's Note

All claims expressed in this article are solely those of the authors and do not necessarily represent those of their affiliated organizations, or those of the publisher, the editors and the reviewers. Any product that may be evaluated in this article, or claim that may be made by its manufacturer, is not guaranteed or endorsed by the publisher.
