# Supplementary material for: Alterations of Oxidative Stress Indicators, Antioxidant Enzymes, Soluble Sugars, and Amino Acids in Mustard [Brassica juncea (L.) Czern and Coss.] in Response to Varying Sowing Time, and Field Temperature
Source: Front Plant Sci. 2022 May 3;13:875009. doi: 10.3389/fpls.2022.875009 (PMC9111527; doi:10.3389/fpls.2022.875009)
Supplement: Supplementary file 1 [file Data_Sheet_1.docx]

Table S1 The physical, mechanical and chemical properties of soil used for experiment during 2017-18 and 2018-19.

| **Soil properties** | **Values** |
| --- | --- |
| Bulk density (g cm^-3^) | 1.49 |
| Particle density (g cm^-3^) | 2.65 |
| Pore space (%) | 43.77 |
| Water holding capacity (%) | 39.46 |
| 1/3 moisture content (%) | 17.83 |
| 1/5 moisture content (%) | 4.57 |
| Coarse sand (%) | 2.38 |
| Fine sand (%) | 55.26 |
| Silt sand (%) | 27.09 |
| Clay (%) | 15.27 |
| Textural classes | Sandy loam |
| Organic carbon (%) | 0.31 |
| Available nitrogen (kg ha-1) | 228.00 |
| Available phosphorus (kg ha-1) | 17.00 |
| Available potassium (kg ha-1) | 180.00 |
| pH | 7.30 |
| Electrical conductivity (ECe; dSm-1) | 0.25 |

Note: Data were obtained from the farm Section of the Institute of Agricultural Sciences, Banaras Hindu University, India.

Table S2 Mean monthly changes in temperature (°C), relative humidity (%) (Morning and evening) and rainfall (mm) of experimental site. Data were taken from 1 November to mid-April (Mid-march) during 2017-18 and 2018-19.

| **Months** | **Relative humidity (%)** | | | | **Temperature (^o^C)** | | | | **Rainfall (mm)** | |
| --- | --- | --- | --- | --- | --- | --- | --- | --- | --- | --- |
|  | **Morning** | | **Evening** | | **Minimum** | | **Maximum** | |  |  |
| Year | 2017-18 | 2018-19 | 2017-18 | 2018-19 | 2017-18 | 2018-19 | 2017-18 | 2018-19 | 2017-18 | 2018-19 |
| November | 90 | 89.17 | 44.15 | 46.63 | 13.50 | 11.95 | 28.08 | 28.55 | 0.00 | 0.00 |
| December | 88.25 | 90.68 | 52.65 | 43.71 | 9.29 | 6.34 | 24.18 | 23.35 | 0.00 | 0.00 |
| January | 92.55 | 89.19 | 57.03 | 52.87 | 6.71 | 6.75 | 20.56 | 21.40 | 0.00 | 17.8 |
| February | 87.07 | 86.57 | 46.21 | 55.79 | 11.58 | 9.69 | 26.35 | 24.31 | 0.00 | 9.80 |
| March | 75.32 | 78.45 | 29.77 | 40.74 | 15.31 | 12.73 | 33.00 | 29.54 | 0.00 | 1.40 |
| April | 64.40 | 69.60 | 33.80 | 31.40 | 20.64 | 19.17 | 35.89 | 37.56 | 9.40 | 0.00 |

Note: Data were obtained from the Institute Meteorology Section of the Department of Agronomy, Banaras Hindu University, India.
