# Supplementary material for: Alterations of Oxidative Stress Indicators, Antioxidant Enzymes, Soluble Sugars, and Amino Acids in Mustard [Brassica juncea (L.) Czern and Coss.] in Response to Varying Sowing Time, and Field Temperature
Source: Front Plant Sci. 2022 May 3;13:875009. doi: 10.3389/fpls.2022.875009 (PMC9111527; doi:10.3389/fpls.2022.875009)
Supplement: Supplementary file 2 [file Image_1.pdf]

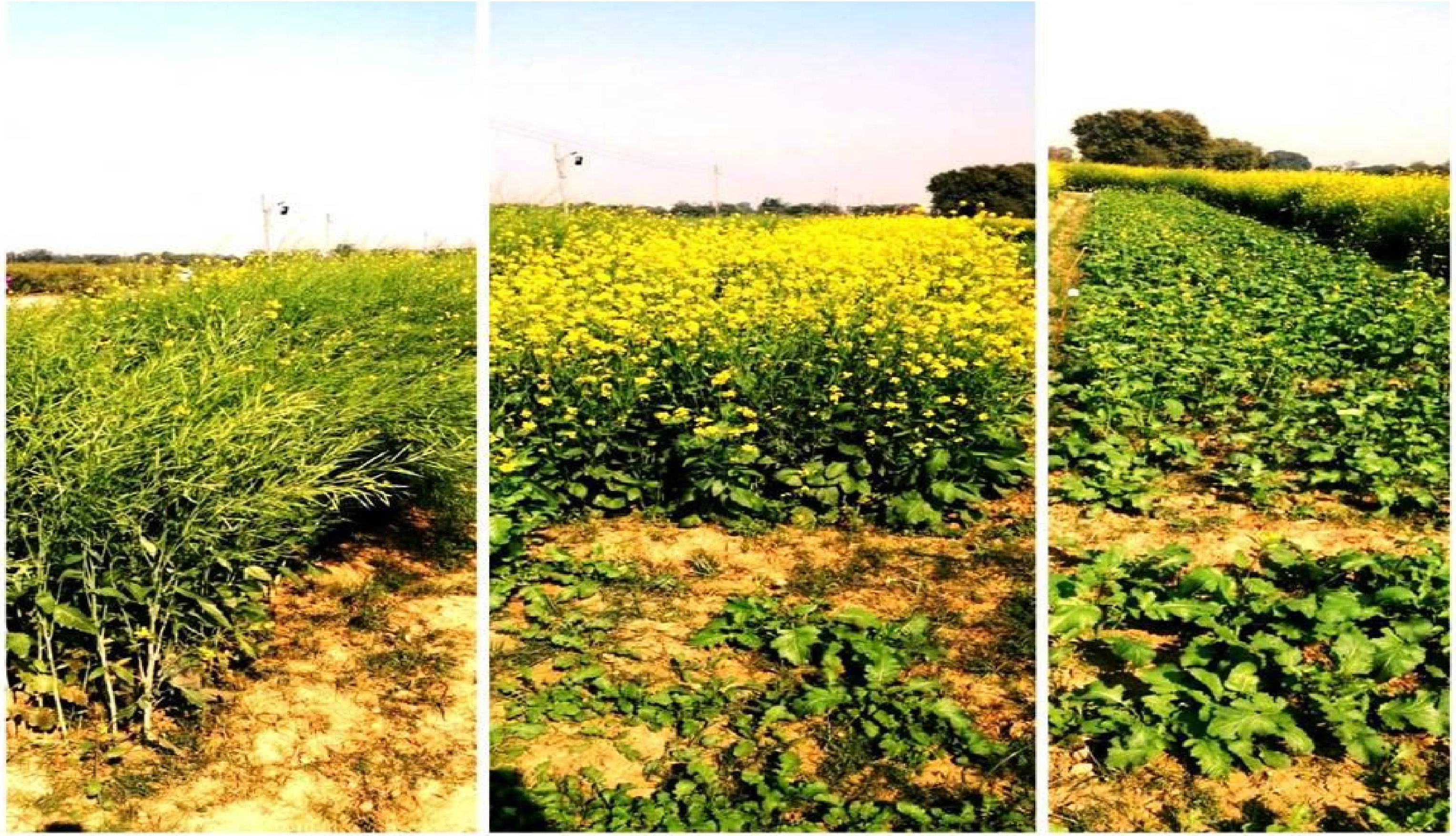

S1 S2 S3

Figure S1 Showed the field view of Indian Brassica at different dates of sowing (S1; 30 October, S2, 15 November, S3; 30 November).
